# Value-based cognitive-behavioural therapy for the prevention of chronic whiplash associated disorders: protocol of a randomized controlled trial

**DOI:** 10.1186/s12891-015-0687-y

**Published:** 2015-09-01

**Authors:** Tonny Elmose Andersen, Sophie Lykkegaard Ravn, Kirsten Kaya Roessler

**Affiliations:** Department of Psychology, University of Southern Denmark, Campusvej 55, DK-5230 Odense M, Denmark

## Abstract

**Background:**

Whiplash injury is the most common traffic-related injury affecting thousands of people every year. Conservative treatments have not proven effective in preventing persistent symptoms and disability after whiplash injury. Early established maladaptive pain behaviours within the first weeks after the injury may explain part of the transition from acute to chronic whiplash associated disorder (WAD). Hence, early targeting of psychological risk factors such as pain catastrophizing, fear-avoidance-beliefs, depression, and symptoms of posttraumatic stress disorder (PTSD) may be important in preventing the development of chronic WAD. Some evidence exists that targeting fear-avoidance beliefs and PTSD with exposure strategies and value-based actions may prevent development of persistent disability after whiplash injury. Yet, the results have to be tested in a randomized controlled trial (RCT). The primary objective of the present study is to test whether a specifically tailored value-based cognitive-behavioural therapy program (V-CBT) is able to prevent the development of persistent disability, pain, and psychological distress if delivered within the first three months after a whiplash injury.

**Methods/design:**

The current study is a two-armed randomized controlled study with a crossover design. Group A is scheduled for V-CBT within one week of randomization and group B with a delayed onset 3 months after randomization.

**Discussion:**

If the study detects significant effects of V-CBT as a preventive intervention, the study will provide new insights of preventive treatment for patients with WAD and thereby serve as an important step towards preventing the chronic condition.

**Trial registration:**

Current Controlled Trials Registration September 19, 2014: NCT02251028

## Background

In Denmark, it is estimated that around 6.000 individuals are exposed to a whiplash trauma every year [1]. Up to 50 % are still experiencing neck pain and disability one year after the incidence [2], and 13 % are disabled to such a degree that they are partly or fully unable to maintain work [3, 4]. Although some evidence exists, that high-intensity strength training may reduce neck pain among industrial workers [5], conservative treatments (analgesics, physiotherapy, and instructions on exercises) have not proven effective in preventing persistent symptoms and disability after whiplash injury [6].

The common recovery process after the injury appears to follow a pattern of rapid improvement within the first three months, with only minor, if any, improvement thereafter [7]. Early established maladaptive pain behaviours within the first weeks after the injury may explain part of the transition from acute to chronic pain. The fear-avoidance model [8, 9] describes how painful stimuli may trigger catastrophic thinking and how fear-avoidance-beliefs ultimately lead to avoidance behaviour, more pain, and depressive thinking. Recently, PTSD has been emphasized as a significant risk factor for the development of chronic pain and disability after a whiplash trauma [7, 10, 11], and a high prevalence of PTSD (15–38 %) has been found among those who have encountered a whiplash trauma [7, 12–14]. PTSD and chronic pain is presumed to maintain one another through an array of cognitive and behavioural mechanisms such as increased hypervigilance, catastrophizing, and avoidance [15, 16]. Hence, early targeting of these psychological risk factors may be important in preventing the development of chronic WAD.

In this regard, promising preliminary support for targeting fear-avoidance behaviour is found in two recent studies successfully treating PTSD [17] and fear of movement in relation to WAD [18]. Both studies achieved clinically significant improvement of PTSD symptoms, pain, and disability. In addition, to exposure-based strategies and CBT techniques, Wicksell and colleagues [19] found that an intervention specifically targeting functional restoration by increasing chronic whiplash injured patients’ willingness to engage in activities in accordance with their life goals and values, significantly improved daily life functioning and life satisfaction compared to a wait-list control group.

Finally, some evidence exists that early intervention, aimed at patients with high levels of perceived disability, pain catastrophizing, and fear-avoidance beliefs, can prevent exclusion from the labour market [20–22]. The greatest success rate of return to work was achieved by intervening between 4 to 12 weeks after the whiplash trauma, indicating that early intervention is important [20]. However, to date no RCT study has used a combined intervention comprising value-based actions and exposure strategies in preventing persistent pain and disability after sub-acute whiplash injury.

### Aims and hypotheses

The aim of the present study was twofold: 1) to test whether a specifically tailored value-based cognitive-behavioural therapy program (V-CBT) delivered within the first three months after whiplash injury can prevent the development of chronic WAD compared to the wait-list controls at 6 months post-randomization and 2) to investigate whether there exists a 3-months window for preventing chronic pain, disability, and psychological distress after a whiplash injury.

### Hypotheses

First, we hypothesize that there will be a significant reduction in the primary and secondary outcomes (disability, pain, and psychological distress) in the V-CBT group (group A) compared to the wait-list controls (group B). Secondly, we hypothesize that the largest effect will be achieved by early intervention within the first 3 months compared with the group receiving treatment after 3 months of waiting. Finally, we expect that these effects will be maintained at a 9 months follow-up post-randomization (12 months post-injury).

## Methods

**Fig. 1:**
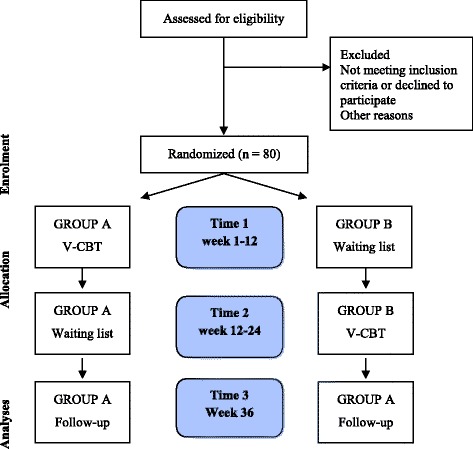
Flow diagram for the procedure

The current study is a two-armed RCT study with a crossover design. Both groups will receive treatment. Group A is scheduled for V-CBT within one week of randomization and group B with a delayed onset 3 months after randomization. See Fig. 1 flow diagram.

### Enrolment

Participants are recruited between 2 to 3 months after the whiplash injury. This is done through the National Patient Register that provides names and addresses on patients who fulfil the ICD-10 diagnose code for a whiplash injury (distorsio columnae cervicalis) and are situated in the region Zealand were the study intervention takes place. Monthly, a research assistant identifies all new whiplash cases in the region Zealand. Potential participants are contacted by letter and invited to participate in the study. All potential participants are asked to rate their average level of pain on a numerical pain rating scales (NRS) ranging from 0 (no pain) to 10 (worst possible pain). Participants who volunteer to participate and experience pain levels corresponding to ≥ 4 on average are further screened by a brief telephone interview. During the telephone screening the participants are orally informed of the project and asked additional questions regarding experienced daily life difficulties in relation to their whiplash trauma. Participants who fulfil the outlined criteria for inclusion (for details, look beneath) and accept participation are referred to one of two trained, clinical psychologist for their first counselling session. Participants are informed that they are allowed to bring a relative to the first consultation. Written informed consent will be obtained from all participants prior to entry into the study. Before the first session and randomization, they receive a letter with additional written information about the study, a statement of consent and the baseline questionnaire. At the first consultation and before randomization, all participants and their relatives are thoroughly informed about the randomization procedure, the intervention, and expected outcome of the study. Participants, who at any given time in the process, do not wish to participate, are not questioned further about this decision.

### Participants

All participants are between the age of 18 and 65-years old and are diagnosed with WAD grade I-II [23]. At 3-months post injury, they are experiencing disability in at least one important life domain (≥5 on the pain disability index) and moderate levels of pain (average pain intensity ≥ 4 on the NRS scale). Also, they have to meet at least one of the psychological risk criteria: elevated levels of pain catastrophizing, fear-avoidance beliefs, symptoms of anxiety and/or depression, and posttraumatic stress symptoms). Clinically relevant cut-off score criteria are based on previous studies [21, 24, 25]. Criteria of exclusion is treatment for other damages sustained in connection with the whiplash trauma, psychiatric disorders, active abuses of alcohol, drugs, or medication, ongoing rehabilitation or other treatment for pain besides physiotherapy and pharmacological treatment, and non-Danish speaking.

### Randomization and blinding

Patients are randomized by random permuted blocks of 4 to 8 by the study statistician at University of Southern Denmark. Randomization is consecutively numbered in sealed opaque envelopes. Patients will randomly be allocated to one of two treatment groups (A or B) in a crossover design with both groups receiving the V-CBT intervention, however with a delayed onset for group B. Group A is scheduled for V-CBT within one week of randomization and group B with a delayed onset of 3-months after randomization. Measurements of effect are carried out at baseline before randomization (3 months post-injury), post-treatment/wait-list (3 months post randomization), and at follow-up (9 months post randomization/12 months post-injury). At all time points patients are asked which potential additional treatment they have received. The clinical psychologists are not blinded to which intervention the patients receive. However, at the point of analysis, the two groups will be coded as X and Y in order to blind the researcher who will conduct analysis.

### Intervention: value-based cognitive-behavioural therapy

The V-CBT is a manualized program specifically tailored for prevention of disability and psychological distress after whiplash injuries [26]. The theoretical foundation of the program is learning theory as outlined by Fordyce [27], and value-based behaviour change strategies as described in contextual cognitive-behavioural therapy [19, 28, 29]. The V-CBT program has a common goal with acceptance and commitment therapy (ACT), to improve daily life functioning and facilitate behaving in accordance with personal values and life-goals. Although, the V-CBT program facilitates behavioural changes according to personal values and life goals, the program does not directly target psychological flexibility by the use of acceptance and mindfulness strategies. The first session of the program is dedicated to assessing personal values and daily life goals. Values and life goals are assessed by an interview based on the patient’s response on the pain disability index, on which affected life domains are reported.

The V-CBT program is rooted in learning theory [27] reflected in the primary focus on imaginal- and in-vivo exposure strategies. For these reasons we have chosen to name the program Value-based Cognitive behavioural therapy. Within the framework of learning theory it is emphasized how negative reinforcement can result in avoidance behaviour and limitations of activity and how events only loosely associated with earlier aversive events may come to serve as cues and conditioned negative reinforces resulting in more avoidance behaviour and activity restrictions. Combining the principles of learning theory with value-based actions, the primary goal of the program is functional restoration of everyday life activities and not necessary symptom eradication. Two trained clinical psychologists deliver the intervention. The psychologists have several years of experience delivering CBT and receive bi-weekly supervision. The program consists of 10 weekly one-hour individual sessions. For a program description, see Table 1.

**Table 1:** Content of the value-based cognitive behavioral therapy program

Session and topic		Aims	Homework/techniques
1-2	Introduction.	To introduce the program. Discuss affected life domains and values. Give insight into the difference between active vs. passive coping.	Discuss important values with the family or a close friend. Complete value template.
	Affected life domains.		
	Values and life goals.		
	Week-plan.		Activity registration.
3	Pain theory and activity engagement according to values.	To introduce pain models and the bio-psycho-social perspective. Discuss values and activity engagement. Setting new short- and long-term value-based goals. Discuss daily activities and make an exercise plan.	Complete week plan according to planned activities.
			Establish a workout routine.
4	“Road-block” therapy.	To discuss psychosocial barriers for fulfilling goals. Introduce the concept negative reinforcement and the power of habits. Introduce the cognitive ABC model. Setting new realistic measureable goals.	Work with barriers, cognitive, emotional or practical.
	Negative reinforcement.		
			Complete week plan according to planned activities.
			Workout.
5	Unfulfilled expectations.	To discuss expectations to oneself and how unfulfilled expectations can affect mood and behaviour resulting in maladaptive coping.	Complete plan B and set realistic goals for the day.
	Having a plan B.		
			Complete week plan according to planned activities.
		Learn to work out a plan B.	
			Workout.
6	The energy balance and psychological barriers for activity engagement.	Learn to conserve “energy”, prioritise in activities.	Identify energy consuming activities vs. activities that increases energy.
		Discuss psychological barriers for activity engagement.	
	Adjust goals.		
			Use the ABC model.
		Discuss life values and adjust goals accordingly.	Complete week plan according to planned activities.
			Workout.
7	Psychological barriers.	To work more in depth with individual psychological distress and barriers for activity engagement, for instance depressive symptoms, PTSD, or pain catastrophizing.	Use the ABC model.
			Complete week plan according to planned activities.
			Workout.
8	Psychological barriers.	To work more in depth with individual psychological distress and barriers for activity engagement. Work out an exposure hierarchy of feared activities.	In vivo exposure.
			Complete week plan according to planned activities.
			Workout.
9	Activity engagement and return to work or relevant activities.	Discuss return to work or relevant activities. Work with fear-avoidance beliefs and catastrophizing.	In vivo exposure of work related activities.
			Complete week plan according to planned activities.
			Workout.
10	Long-term goals and values.	To discuss progress and values. Set long-term goals and work out a plan for setbacks and maintenance of progress.	Discuss long-term goals and plan with a close relative or friend.

Before the first session, all patients receive a 14-min psycho-educational video produced with the purpose of reassurance after acute and subacute whiplash injury. The video includes education in the physiology of a cervical strain, possible symptoms and prognosis, coping strategies, and encouragement to gradually maintain pre-injury activity.

The main components of the V-CBT program are: 1) Education in pain theory and active coping, 2) unlearning fear-conditioned movement restrictions, 3) gradual increase in activity, 4) daily program of movement or excises, 5) change of dysfunctional thoughts and catastrophizing and focused treatment of mild PTSD-symptoms by imaginal and in-vivo exposure, and 6) development of a plan on how to return to work or relevant activities. The patients are expected to participate actively between sessions by doing homework. Weekly goals regarding resumption of daily activities or tasks are set throughout the program.

### Measures

#### Primary outcome

The primary measure of outcome is disability as measured with the Pain Disability Index (PDI) [30]. The PDI measures how pain interferes with daily life activities within 7 different domains. The 7 domains are rated from 0 (no disability) to 10 (worst disability). The scale shows good reliability and validity [31].

#### Secondary outcomes

Neck pain intensity and disability is measured with the Neck Disability Index (NDI) [25]. The NDI measures within 10 domains how neck pain affects the ability to handle daily life activities such as personal care, lifting, reading, work, driving, sleeping, recreational activities, pain intensity, concentration, and headache. The total score range from 0 (no disability) to 100 (total disability).

Pain is also measured on four numerical pain rating scales (NRS) ranging from 0 (no pain) to 10 (worst possible pain). Patients mark their answers on each scale corresponding to their pain now, highest level of pain, lowest level of pain, and finally average pain over the past week (NRS) [32].

Fear of re-injury due to movement is measured with Tampa Scale for Kinesiophobia (TSK) [33]. TSK is a 17-item scale assessing fear of movement on a 4-point likert scale ranging from 17 to 68 with higher scores indicating higher levels of kinesiophobia. The scale is commonly used in various chronic pain samples and has good construct and predictive validity [34].

Catastrophic thinking related to pain is measured with the Pain Catastrophizing Scale (PCS) [35]. The PCS ask participants to reflect on past painful experiences and to indicate the degree to which they experienced each of 13 thoughts or feelings when experiencing pain on a five-point Likert scale with (0 = not at all, 4 = all the time). The PCS has been shown to have high internal consistency and to be associated with heightened pain and disability [35]. A high score indicates high level of pain catastrophizing.

To assess the level of anxiety and depressive symptoms, the Hospital Anxiety and Depression Scale is used (HADS) [36]. The scale consists of 14 items, seven relating to anxiety (HADS-A) and seven to depression (HADS-D) with responses ranging from 0 (no symptoms) to 3 (maximum impairment). Internal consistency is high and the scale has proven sensitive for detecting clinical changes [37].

PTSD symptomatology is measured with the PTSD-8 [24]. The scale is a brief version of The Harvard Trauma Questionnaire part IV [38]. The PTSD-8 consists of 8 items on a four-point Likert scale (1 = not at all, 4 = very often). The items relate to the three core clusters in PTSD in DSM-IV: avoidance (2 items), intrusion (4 items), and hyperarousal (2 items). The scale has proven good psychometric properties in various trauma samples including whiplash injured [24].

### Sample size and statistical analysis

To our knowledge no similar studies have been conducted. For this reason, the power calculation is estimated according to the earlier studies using CBT and acceptance-based strategies with functional restoration and activity engagement as their primary goal. From these earlier results, a moderate effect size is to be expected (η_p_^2^ = 0.25) [19, 20]. By putting the level of significance at 5 %, the power at 80 %, and the expected dropout at 9 months post-randomization to be 10 %, it is calculated that there should be 40 patients in each group (A & B).

The primary and secondary outcomes measured for both groups at baseline before randomization (3 months post-injury), 3 months post-randomization, and 9 months post randomization (12 months post-injury) will be analysed using linear mixed-effects models (random coefficient models and multilevel models). With the mixed effects model approach all available data will be used and intention-to-treat analyses applied.

### Ethics statements

The study is presented and approved of The Regional Scientific Ethical Committee for Southern Denmark (J.nr. S-20130103) and the Danish Data Protection Agency. All procedures in the study are in accordance with the second declaration of Helsinki. The intervention is an additional offer to treatment as usual, and everybody is free to say no to participation.

## Discussion

The primary objective of the present study is to test whether the specifically tailored V-CBT program is able to prevent the development of persistent disability, pain, and psychological distress if delivered within the first three months after a whiplash injury. In particular, early identification and targeting of fear-avoidance beliefs, pain catastrophizing, and symptoms of PTSD seem to be of importance.

In contrast to symptom reduction, aiming to improve functioning and activity engagement in patients has shown promising results [18, 19, 29]. In particular, the combination of exposure and value-based strategies for functional restoration may be important. Also, addressing PTSD symptomatology seems essential in relation to functional restoration [17]. Based on learning theory [9, 27], maladaptive pain behaviours may develop within the fist weeks after an injury, why early intervention may prevent this maladaptive pattern. Indeed, this is indicated by Adams and Colleagues [20], finding their intervention most effective when delivered within three months after the whiplash injury. However, research has yet to determine in a RCT study whether targeting these early risk factors will prevent long-term disability and psychological distress. Also, it is important to gain knowledge whether there exists a 3-months window for intervention, as indicated by longitudinal cohort studies of recovery after whiplash injury [7].

Whiplash injury is the most common injury after traffic accidents and represents a major societal and personal problem. For this reason, if effective, the intervention program will have significant importance for both patients and the society. Moreover, the program can easy be implement in already established pain clinics around the country. In the present study no health economic analyses are planned. However, recently a study by Kemani and colleagues [39] showed that acceptance and commitment therapy for longstanding pain was more cost-effective than an active control treatment. The present study could be strengthened by a register study of the long-term health-economic effects of the V-CBT program.

The study can be subjected to some challenges. It may be difficult to recruit patients in the sub-acute phase since most patients are informed that their symptoms will disappear by themself. Also, most patients hold a traditional biomedical view of the injury, why an early psychological intervention seldom is their first choice of treatment. Also, dropout could be significant due to remission or change in priorities during the intervention. In order to prevent this, motivational factors are kept in mind during the process of treatment. Patients enrolled in the study are contacted by phone before onset of treatment in order to remind them and motivate them for coming. Also, at follow up, patients are reminded personally. Finally, it is important to keep in mind, that although the intervention may prove effective in preventing the development of chronic WAD, the intervention is aimed at the particular subgroup of WAD with a psychological risk profile. Not all WAD are associated with pain related anxiety, and avoidance behaviour needing treatment [40].
